# Meaningless but memorable: Reward associations boost recognition of abstract visual stimuli

**DOI:** 10.3758/s13415-025-01387-w

**Published:** 2026-03-16

**Authors:** Francesco Grassi, Esther A. Semmelhack, Anne Schacht

**Affiliations:** 1 Department of Cognition, Emotion and Behavior, University of Göttingen, Gosslerstrasse 14, 37073 Goettingen, Germany; 2 CRC 1528 ‘Cognition of Interaction’, University of Göttingen, Goettingen, Germany

**Keywords:** Associative learning, Motivational relevance, Memory, Event-related brain potentials, Pseudowords

## Abstract

Visual word processing engages perceptual and memory-related mechanisms that allow rapid identification of written stimuli. While most research relies on meaningful words, recent work suggests that sublexical visual features—such as character strings—can similarly engage neural processes related to attention and memory. The present study investigated how motivational relevance and the memory status of the stimulus interact to shape recognition memory, as reflected in P300/LPC amplitude modulations. Across two event-related brain potential experiments (*N* = 48), participants learned associations between meaningless consonant strings and monetary outcomes (gain, loss, or zero outcome) based on either the character combination (Experiment 1) or the font type (Experiment 2). In a subsequent old/new recognition task, we orthogonally manipulated memory status and relevance by presenting both previously learned and novel stimuli. Across both experiments, we observed robust old/new effects in the P300/LPC time window. Critically, gain-old stimuli elicited enhanced P300/LPC amplitudes and higher recognition accuracy—indicating that motivational relevance can strengthen recollection even in the absence of meaning. In Experiment 2, this neural enhancement diminished over time, suggesting a dynamic shift from relevance-driven to familiarity-based memory processes when reinforcement is no longer active. These findings suggest that motivational relevance alone is sufficient to enhance memory performance and neural processing for abstract, nonsemantic stimuli. They also underscore the importance of low-level visual features in sublexical stages of visual word recognition and highlight how the impact of motivational associations may change as recognition strategies adapt over time.

**Supplementary Information:**

The online version contains supplementary material available at 10.3758/s13415-025-01387-w.

## Introduction

Visual word processing is a fundamental cognitive function that enables humans to recognize and comprehend written language. This capacity allows for the rapid extraction of abstract structural properties and meaning from complex visual stimuli that can vary in size, font, and retinal position (Rayner & Pollatsek, 2013). The efficiency of visual word recognition is particularly remarkable given that reading is a relatively recent cultural invention, suggesting a high degree of neural plasticity in adapting existing visual and language systems for this purpose (Baker et al., 2007; McCandliss et al., 2003). Many cognitive models of visual word processing emphasize the central role of lexical access and semantic networks in guiding attention, memory, and decision-making. However, less is known about the extent to which purely perceptual or associative factors—such as visual feature learning or motivational conditioning—can shape recognition processes independently of semantic content. While much of the existing work on visual word recognition has relied on behavioral measures using real words to study lexical access and semantic activation, electrophysiological methods, such as event-related brain potentials (ERPs) offer fine-grained insights into the temporal dynamics of these processes.

Event-related brain potentials are time-locked neural responses to specific sensory, cognitive, or motor events and have provided valuable insight into the brain’s processing stages during visual word recognition. Several ERP components correspond to distinct stages of word recognition, lexical access, and memory retrieval. For instance, encoding-related components include the P1 and N1, reflecting early visual and orthographic processing, while later components, such as the P300 and Late Positive Complex (LPC), are linked to higher-order processes, including attention, memory, and the evaluation of motivational significance (Polich, 2007; Schupp et al., 2006). These later components are not specific to word processing but have been broadly associated with elaborative and evaluative processing across a range of visual and cognitive tasks. In visual word processing paradigms, the P300 and LPC have been tied to recollection-based memory mechanisms and the processing of emotional and motivational significance. It is important to note, however, that while the P300 and LPC have sometimes been discussed as distinct components in the literature, many studies involving memory or motivational/emotional paradigms have labeled centroparietal positivities occurring roughly between 300 and 600 ms after stimulus onset either as P300 or LPC (Bayer et al., 2019; Friedman, 1990; Grassi et al., 2024; Rossi et al., 2017; Rugg & Nagy, 1989) or have not clearly differentiated between them (Curran, 2000; Nessler et al., 2001; Rugg & Curran, 2007). In this context, the term Late Positive Potential (LPP) is also frequently used, especially in affective and motivational paradigms (Schupp et al., 2006).

Aside from the specificity of both components, research has consistently demonstrated that modulations in the P300/LPC complex reflect (episodic) recollection-based recognition memory processes. Specifically, this centroparietal positivities exhibit enhanced amplitudes when participants correctly recognize previously encountered words, in contrast to novel stimuli, a phenomenon commonly referred to as the "old/new effect" (Curran, 2000; Friedman, 1990; Nessler et al., 2001; Rugg & Nagy, 1989; for a review, see Rugg & Curran, 2007).

In addition to reflecting memory-related processing stages, the P300/LPC is sensitive to the emotional and motivational relevance of stimuli. Words that carry emotional meaning, particularly positive words, tend to evoke larger P300/LPC amplitudes compared with neutral words (Citron, 2012; Hinojosa et al., 2010; Schacht & Sommer, 2009). This heightened response likely reflects increased attentional allocation and deeper cognitive engagement with emotionally relevant stimuli, which can in turn strengthen memory encoding and retrieval. Indeed, there is substantial evidence that emotionally arousing events and stimuli are remembered more vividly and persist for longer periods (Bradley et al., 1992; Dolcos et al., 2005; Weymar et al., 2009). Accordingly, it is not surprising that the emotional relevance of a word can amplify old/new effects on the P300/LPC, resulting in greater component amplitudes in response to familiar emotional words (Dietrich et al., 2001; Inaba et al., 2005; Weymar et al., 2013). This interaction suggests that emotional relevance enhances word recollection, with the P300/LPC serving as a marker of the combined influence of emotional relevance and memory status (i.e., whether a word is old or new).

Another line of research has attempted a more focused investigation into how motivational relevance interacts with early orthographic and perceptual processes within the visual word recognition system. When real words are used as stimuli, the automatic activation of semantic and phonological networks can obscure these low-level processes. To circumvent this potential confound, several studies have employed pseudowords or other meaningless symbolic stimuli (Bayer et al., 2019; Fritsch & Kuchinke, 2013; Grassi et al., 2024; Kuchinke et al., 2015; Kulke et al., 2019; Rossi et al., 2017). Despite their lack of semantic content, such stimuli still engage key subcomponents of visual word processing. Indeed, prior research has consistently demonstrated that letter strings—irrespective of lexical status—activate early orthographic and perceptual processes within the visual word recognition system (Dehaene et al., 2005; Grainger & Holcomb, 2009). Adopting this broader definition of visual word processing, which encompasses the extraction of visual-orthographic patterns from novel or unfamiliar stimuli, reflects a deliberate focus on these core sublexical mechanisms without invoking semantic dimensions.

In these studies, stimuli acquired motivational relevance through associative learning, typically by being associated with positive or negative monetary outcomes (referred to here as “associated relevance,” i.e., motivational value tied to specific stimuli, not general task-induced motivation). This distinction is important, as performance-contingent rewards can increase general task engagement or cognitive control (Engelmann, 2009; Esterman et al., 2014; Jimura et al., 2010; Locke & Braver, 2010), whereas our study focuses on stimulus-specific learned associations formed during encoding. By isolating such item-specific associations, these studies aimed to determine whether motivational relevance alone—independent of semantic content—can modulate ERP responses similarly to meaningful emotional words (cf. Kulke et al., 2019, for a direct comparison between words and associated pseudowords). Findings from these studies show that, following an associative learning phase, old stimuli exhibit enhanced P300/LPC amplitudes compared with new stimuli, mirroring the old/new effects observed with real words. However, unlike studies with meaningful words, these studies do not show an additional interaction between motivational relevance and stimulus old/new status in modulating the P300/LPC. This absence of interaction raises questions about the necessity of semantic content for the observed memory improvements associated with motivational relevance. In other words, the emotional relevance derived from the semantics of real words may be essential for the enhanced P300/LPC responses observed during memory recognition. However, alternative considerations challenge this interpretation. First, evidence suggests that the motivational relevance associated with symbolic stimuli can influence multiple stages of stimulus processing, similar to real words with emotional content. Previous studies have observed modulations in various ERP components, as early as the first 100 ms after stimulus presentation, and also including the P300/LPC (Bayer et al., 2019; Grassi et al., 2023, 2024; Rossi et al., 2017; Schacht et al., 2012). This indicates that motivational relevance can be associated to the low-level visual features of the stimuli, thereby enhancing neural processing even in the absence of semantic meaning. Second, many of these studies (Bayer et al., 2019; Grassi et al., 2023; Kulke et al., 2019) have shown that pseudowords with associated motivational relevance are recognized more quickly and accurately, providing behavioral evidence that motivational relevance can enhance memory performance. Methodological factors may also explain the lack of interaction effects reported in the literature. First, many memory tests did not require participants to attend to or evaluate the motivational relevance previously learned during the association phase—that is, the specific outcome values tied to each stimulus—rather than general task goals. This may have reduced the salience of motivational relevance during retrieval and led to its diminished neural effects. Second, novel stimuli were often repeated across trials, gradually reducing their novelty and weakening the old/new contrast in ERP amplitudes. Third, most designs did not independently manipulate motivational relevance and stimulus familiarity (old/new); instead, old stimuli were motivationally relevant and new stimuli were neutral. This confound made it difficult to disentangle the unique and combined effects of relevance and familiarity. Finally, the visual features used to encode motivational associations—such as font or character identity—were often not counterbalanced or systematically varied, which may have introduced perceptual biases that obscured interaction effects (Grassi et al., 2023, 2024). Taken together, these design limitations suggest that the absence of interaction effects in prior work should not be taken as strong evidence that semantic content is required for motivational enhancement of memory-related ERP responses.

The current study aims to address this open question by employing a design that orthogonally manipulates motivational relevance and memory status of symbolic stimuli in the absence of semantic content, to clarify their individual and combined effects on neural processing as measured by the P300/LPC component. By doing so, we test whether semantic meaning is a necessary condition for motivational modulation of memory-related brain responses or whether such effects can be driven by learned value associations linked solely to low-level visual dimensions. This addresses a broader theoretical question in memory and motivation research: Can nonsemantic visual stimuli elicit prioritization in memory encoding and retrieval when motivationally relevant?

The study builds on ERP measures from the test session of Grassi et al. (2023). It employed a between-subjects design consisting of two parallel experiments (*N* = 24 each). In both experiments, meaningless strings of consonants acquired motivational relevance during a learning session by being associated with different monetary outcomes: gain, loss, or zero outcome. The two experiments differed in the dimension of the low-level visual features that were relevant for the association, namely the characters or the font of the stimulus. In that study, ERP analysis of the learning phase revealed increased LPC amplitudes for gain-associated (and to a lesser extent for loss-associated) stimuli compared with those for zero outcome ones in the 300- to 600-ms time window. Notably, LPC amplitudes for gain-associated stimuli consistently increased compared with zero outcome stimuli throughout the learning phase of both experiments. Following the learning phase, participants completed a test session in which they performed an old/new decision task. Importantly, the new stimuli in this task shared the same dimension of low-level visual features that had previously been associated with monetary outcomes but differed in the other visual dimension. This design enabled the orthogonal manipulation of motivational relevance and stimulus memory status, allowing a clear assessment of their individual and combined effects on P300/LPC component amplitudes. To avoid terminological ambiguity, we refer to “memory status” strictly as an experimental manipulation (i.e., whether a stimulus is new or previously encountered) and to “old/new ERP effects” when describing corresponding neural modulations in the P300/LPC time window. To improve clarity and flow, we emphasize that visual word recognition is typically studied using meaningful linguistic stimuli (e.g., real words), which inherently engage lexical and semantic systems. By contrast, our use of abstract consonant strings allows us to examine sublexical stages of processing and their sensitivity to learned value associations. This enables us to explore whether motivational prioritization in memory can emerge from perceptual and associative mechanisms alone, independent of semantic networks—an issue that bears directly on theories of reward-modulated encoding and attention–memory interactions in both linguistic and nonlinguistic domains.

The primary goal of the study was to clarify how the interaction between motivational relevance and memory status impacts neural responses, as reflected in P300/LPC amplitudes. Based on prior evidence of enhanced memory and ERP responses for positively reinforced symbolic stimuli (Grassi et al., 2023; Rossi et al., 2017), we hypothesized that the P300/LPC amplitudes would be particularly enhanced for old stimuli previously associated with monetary gain. While exploratory analyses considered all outcome categories (gain, loss, and zero outcome), our strongest a priori prediction concerned the preferential processing of gain-associated stimuli**,** reflecting a positivity bias in associative learning and memory (Bayer et al., 2019; Ferré, 2003; Hammerschmidt et al., 2017, 2018; Schacht et al., 2012). The secondary goal was to investigate how the interaction effect of associated relevance and stimulus old/new status would evolve over the course of the recognition task. This prediction is grounded in prior findings from associative learning paradigms showing that, in the absence of reinforcement, the influence of previously learned associations on memory performance and neural signatures tends to decay over time (Schott et al., 2007; Wimmer & Shohamy, 2012). We hypothesized that P300/LPC modulations would diminish over time owing to 1) extinction of the learned associations in the absence of reinforcement, and/or 2) increased familiarity with the novel stimuli, thereby reducing the old/new contrast. Finally, our design allowed for the exploration of potential differences in the effects of motivational relevance and familiarity based on the specific low-level visual features—whether characters or font—that were associated with relevance.

## Methods

### Participants

Data were collected from 50 female participants. The sample size was determined based on the guideline of including at least ten times the number of predictors used in the statistical analysis. There were no significant differences between the two groups that could account for behavioral or neural differences. All participants were native German speakers, right-handed, had normal or corrected-to-normal vision, and had no history of neuropsychological disorders. Most participants were psychology students at the University of Goettingen and the PFH Goettingen. Two participants were excluded owing to insufficient learning success, defined as performance not exceeding chance level (33%) in at least one outcome category during the final quarter of the learning session. This criterion ensured that only participants who had reliably learned the stimulus-outcome associations were included in the analyses. This left 24 participants in each of the two experimental groups. In the first group, the participants’ age ranged from 19 to 31 years (*M*_*age*_ = 22.58, standard deviation [*SD*] = 2.7), and in the second group, ages ranged from 19 to 29 years (*M*_*age*_ = 22.42, *SD* = 2.3). Participants were compensated either €8.50 per hour or received course credit, in addition to a performance-based monetary bonus.

### Stimuli

**Fig. 1:**
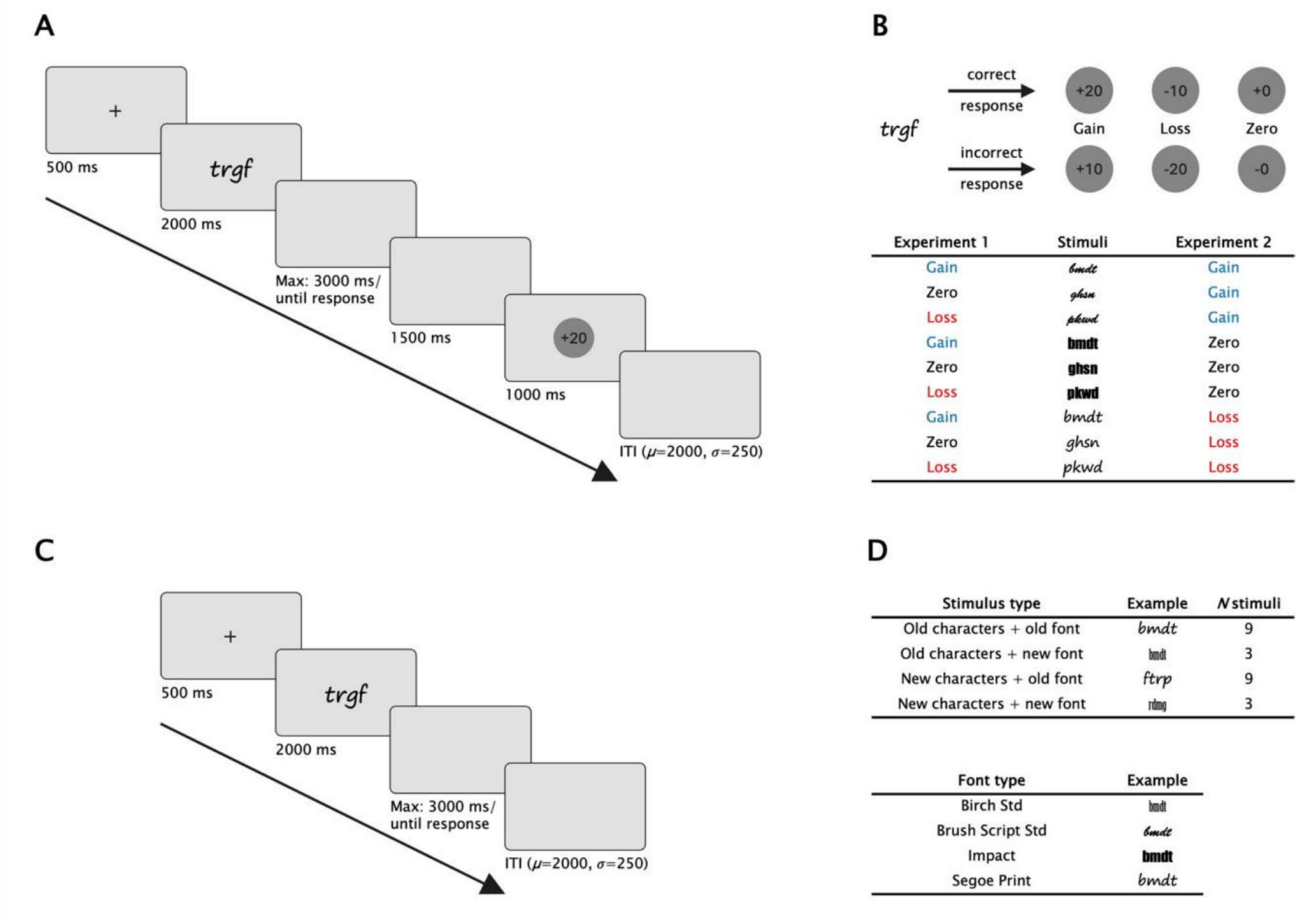
Scheme of the experimental procedure, as described in Grassi et al. (2023). (**A**) Learning session. (**B**) Top: feedback stimuli in accordance with the different outcome conditions and participant response. Bottom: assignment of stimuli to the outcome conditions in accordance with the relevant feature of association of each experimental group. (**C**) Test session. (**D**) Top: scheme of the manipulation of the stimuli’s low-level visual features. Bottom: font types used in the study and example stimuli

Fifteen unpronounceable letter strings, each consisting of four consonants without repetition (e.g., “pdst”; “mfhr”), were used as stimuli. These strings were created in MATLAB (R2018a), with luminance adjusted to ensure consistency. The letter strings were presented in four different fonts: Birch Std, Brush Script Std, Impact, and Segoe Print (Fig. 1D). These fonts were chosen based on a pilot study in which 20 fonts were rated, with the four chosen fonts being identified as the most dissimilar.

### Procedure

The study was approved by the local ethics committee of the Institute of Psychology at the University of Göttingen. Experimental sessions were conducted in an EEG cabin. Participants were seated in height-adjustable chairs with their chin resting on an adjustable chin rest. The experiment consisted of two sessions: a learning session and a test session, with the latter conducted the following day. The experiment was programmed in Python (version 3.8.6) using the PsychoPy package (version 2020.2.0; Peirce et al., 2019) and run via PyCharm (version 2017.2.3). All stimuli were displayed centrally on a BenQ XL2411Z monitor positioned 60 cm away from the participant. The screen resolution was 1920 × 1080 pixels, with a refresh rate of 60 Hz. Stimuli subtended a visual angle of approximately 1.8° in height and 4.5° in width. Font size was standardized across all conditions to ensure consistent perceptual salience across fonts.

#### Learning session

After providing informed consent, participants were presented with a total of nine different stimuli, consisting of three of the 15 letter strings, each presented in three of the four fonts. Both the letter strings and fonts were randomly selected for each participant. The stimuli were associated to one of the three monetary outcomes: gain, loss, or zero outcome. The key distinction between the two experimental groups was the dimension of the low-level features that was relevant for the associations:

##### **Experiment 1: Character-association group**

The combination of consonants in each string determined the outcome condition, with the font being irrelevant. The same letter string was always associated with a specific monetary outcome, independent of the font type (Fig. 1B).

##### **Experiment 2: Font-association group**

The font type was used to assign the stimuli to the different outcome conditions. Each font was associated with either a gain, loss, or zero outcome. Therefore, participants had to focus on the font type and disregard the letter strings to correctly assign the stimuli (Fig. 1B).

In both experiments, participants learned the associations between the stimuli and their corresponding outcome category by pressing one of three arrow keys on a standard German keyboard. The left and right arrow keys were used for gain- and loss-associated stimuli, counterbalanced across participants, while the down arrow key was assigned to the zero outcome condition. A trial scheme of the learning session is shown in Fig. 1A. At the beginning of the session, participants received written instructions about the task and the association paradigm, but no prior information about the outcome categories was given. Participants had to learn the stimulus-outcome associations by trial and error. Feedback indicating the correctness of each decision and the associated monetary outcome was provided after each trial. Participants started with a balance of €3. Each trial began with a fixation cross displayed in the center of the screen for 500 ms, followed by a letter string that remained on the screen for 2,000 ms. If no response was made during the presentation, a blank screen was shown for a maximum of 3,000 ms or until a response was made. This was followed by another blank screen for 1,500 ms. Feedback was presented on a light grey disk for 1,000 ms, indicating the amount of money gained or lost in the trial and whether the correct key was pressed. The feedback also indicated the outcome category to which the stimulus belonged.

Participants earned 20 cents for correctly categorizing a gain-associated stimulus, while an incorrect categorization resulted in a 10-cent earning. For loss-associated stimuli, participants lost 10 cents if they pressed the correct response and 20 cents for an incorrect response. For zero outcome stimuli, a “ + 0” or “ − 0” indicated a correct or incorrect response, respectively. Failure to respond within 5,000 ms after stimulus onset resulted in a 50-cent loss. This higher penalty for nonresponses was intended to incentivize timely decisions, particularly early in the learning session when participants were still forming associations. Importantly, no participant exhibited a systematic pattern of response omissions for any specific outcome category, making it unlikely that missed trials introduced confounds in the acquisition of stimulus–outcome associations. After a randomly jittered inter-trial interval between 1,750 and 2,250 ms, the next trial began. The learning session consisted of 40 blocks, with the nine stimuli presented in random order in each block, leading to a total of 360 trials. The entire session lasted approximately 60 min, with a break after the first half of the trials. During the break, the participant’s current balance was displayed on the screen.

#### Test session

The test session took place one day after the learning session to allow for overnight memory consolidation. Participants were presented with the nine previously learned letter strings, mixed with novel stimuli, and were required to perform an old/new decision task. The novel stimuli consisted of three types: (a) old letter strings paired with new fonts, (b) new letter strings paired with old fonts, and (c) completely new letter strings with new fonts (Fig. 1D). Participants were not informed that novel stimuli could share features (e.g., characters or fonts) with previously learned items. They were instructed to base their judgments on whether the exact stimulus configuration had been encountered during the learning session. This omission was intentional to ensure that recognition judgments reflected holistic familiarity rather than dimension-specific recall. The test session consisted of 40 blocks, each containing 24 stimuli (9 old and 15 new), totaling 960 trials. Each trial was separated by a randomly jittered inter-trial interval between 1,750 and 2,250 ms to prevent temporal anticipation. Participants responded by pressing either the left or right arrow key, with counterbalanced assignment. No feedback was given during the test session.

### EEG recording and preprocessing

EEG recordings were made during both sessions using 64 active Ag–AgCl electrodes mounted in an electrode cap (Easy Cap) according to the extended 10–20 system (Pivik et al., 1993). External electrodes were placed on the mastoids bilaterally, as well as on the outer canthi (HEOG) and below the eyes (VEOG) to monitor ocular movements. The common mode sense (CMS) active electrode was used as the reference, and the driven right leg (DRL) passive electrode served as the ground. The EEG signals were amplified using a Biosemi ActiveTwo AD-box (24 bits; band-pass filter 0.16–100 Hz) and recorded using the ActiView software at a sampling rate of 512 Hz. Electrode offsets were maintained within ± 20 mV.

EEG data were preprocessed using EEGLAB (version 2021.1; Delorme & Makeig, 2004), ERPLAB (version 8.10; Lopez-Calderon & Luck, 2014), and BioSig (version 3.7.9; Schölgl et al., 2011) toolboxes in MATLAB (R2018a). Signals were re-referenced to the average of the mastoid electrodes. High-pass (0.1 Hz) and low-pass (40 Hz) filters were applied, and the timing of triggers was adjusted by shifting them by 25 ms to account for the delay between stimulus presentation and EEG recording. Epochs were segmented to include 1.2 s around the stimulus onset, with baseline correction performed using the 200 ms pre-stimulus period.

Independent Component Analysis (ICA) was employed to remove ocular artifacts, blinks, and channel noise. One channel from one participant in the learning session and one from another participant in the test session were interpolated using spherical interpolation due to poor signal quality. Noisy trials were excluded if voltage values exceeded ± 100 µV or if the slope exceeded 100 µV within the epoch. Trials with improbable values greater than 3 standard deviations from the mean probability distribution (at both the channel and whole-brain levels) were also removed. All trials were visually inspected to confirm data quality. On average, 18.38% of trials were rejected in the learning session of Experiment 1 (range 5.56% to 44.44%), and 25.15% were rejected in Experiment 2 (range 8.61% to 47.22%). In the test session, 22.31% (range 10.42% to 37.60%) and 22.06% (range 7.08% to 36.25%) of trials were rejected in Experiments 1 and 2, respectively. All channels were re-referenced to the average following preprocessing.

### Data analysis of the test session

The data analysis focused on the behavioral responses and ERP amplitudes from the test session, separately for the two experiments.

#### Behavioral analysis

Participant response in the old/new decision task was analyzed by determining the posterior distribution (coefficient p of a Bernoulli distribution) for the probability to attribute memory status category correctly, using a Bayesian additive multilevel regression model for binomial response (Fahrmeir et al., 2009). The model was implemented in the Bayesian statistical software BayesX (version 3.0.2; Belitz et al., 2015) with the mcmcreg object. A Markov Chain Monte Carlo algorithm with 52,000 iterations, a burn-in period of 2000, and a thinning parameter of 50 was employed to fit the model. The input data for BayesX included the number of correct trials per block, along with relevance and memory status category. To account for the dependence of the performance of one participant across blocks, varying intercepts and slopes were included for participants across time. The alpha and beta priors were weakly informative (α = 0.001, β = 0.001), because no strong assumptions about the prior distribution could be made. To assess statistical significance, we applied a criterion based on nonoverlapping 99% credible intervals between the posterior distributions of the relevance conditions. Pairwise differences were considered significant at time points (i.e., blocks) where these intervals did not overlap. This analysis was performed separately for each of the two memory status conditions (old and new), allowing us to examine how behavioral performance evolved as a function of motivational relevance over time.

#### Mass univariate analysis

Given the variability in the temporal and spatial localization of ERP old/new effects in previous literature (Bayer et al., 2019; Curran & Hancock, 2007; Dietrich et al., 2001; Herbert et al., 2008; Inaba et al., 2005; Kissler et al., 2009; Kulke et al., 2019), our approach included a first data-driven step to ascertain the presence of old/new effects, irrespective of any modulation by associated relevance. Once such effects were confirmed, subsequent analyses tested for interactions with relevance within the spatiotemporal extent of the identified clusters. The first step involved testing for stimulus memory status effects (old vs. new) on P300/LPC amplitudes using a Factorial Mass Univariate Analysis with permutation-based correction for multiple comparisons (Groppe et al., 2011). Old stimuli were defined as those presented during the learning session. New stimuli differed across experiments: in the character-association group, they consisted of the same characters in novel fonts; in the font-association group, they consisted of new character strings presented in previously used fonts (Table 1).

**Table 1.**
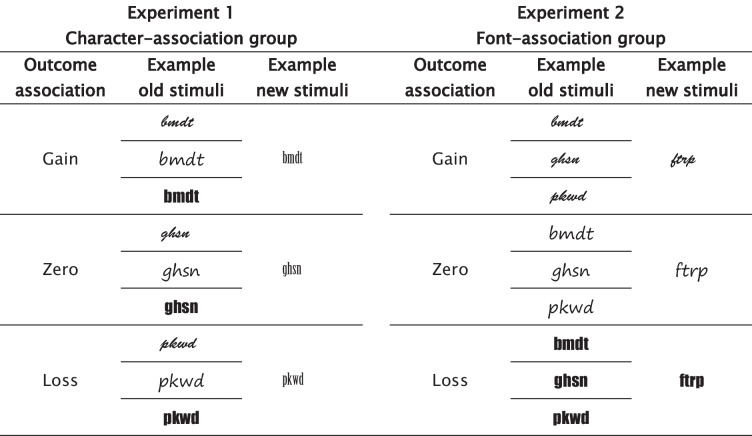
Assignment of stimuli to the outcome and old/new categories during the test session

Category-assignment done in accordance with the relevant feature of association of each experimental group. (Left): Experiment 1; outcome association based on the set of characters; new stimuli obtained by changing the font. (Right): Experiment 2; outcome association based on font; new stimuli obtained by changing the set of characters

To enhance statistical power, we restricted the analysis to a predefined spatiotemporal ROI encompassing the typical P300/LPC window (ROI; Fields & Kuperberg, 2019). Based on prior literature, this ROI spanned 300–600 ms and included centroparietal electrodes: Pz, POz, P1, P2, P3, P4, PO3, and PO4 (Bayer et al., 2019; Herbert et al., 2008; Inaba et al., 2005; Kissler et al., 2009; Kulke et al., 2019).

The analysis was conducted using MATLAB (R2018a), with EEGLAB (version 2021.1), the Mass Univariate ERP toolbox (Groppe et al., 2011), and the Factorial Mass Univariate ERP toolbox (Fields, 2017). An ANOVA was computed at each time point and electrode within the predefined ROI, with stimulus memory status (old/new) as the main factor. Spatial–temporal clusters of significance were formed by grouping adjacent electrodes and time points exceeding a threshold of *p* <.01. The cluster “mass” was defined as the sum of F-values within each cluster.

To assess statistical significance while controlling for multiple comparisons, a nonparametric permutation test was used. Condition labels were randomly shuffled within each participant, and the maximum cluster mass was recalculated for 50,000 permutations to generate a null distribution. Observed clusters were deemed significant if their cluster mass exceeded the 95th percentile of the permutation distribution, corresponding to a family-wise α-level of.05.

Importantly, Factorial Mass Univariate Analysis does not yield conventional model coefficients or standard errors, because it is a nonparametric technique. Instead, cluster-level *p*-values and cluster mass values provide the appropriate inferential metrics and are used throughout our results to evaluate old/new and interaction effects within the ERP data.

#### ERP analysis

In a second step, the interaction effect of stimulus memory status and associated relevance on P300/LPC amplitudes was tested, as well as how this interaction evolved across trials in the test session. Once a significant cluster was identified within the predefined ROI, mean ERP amplitudes across its spatial and temporal extent were extracted for each trial and participant.

Linear Mixed Models (LMMs; Baayen et al., 2008) were then used to assess the interaction effects of stimulus memory status, associated relevance, and trial number on the extracted mean amplitudes. Analyses were performed using R (version 4.1.0; R Core Team, 2020) with the function *lmer* from the *lme4* package (version 1.1–27; Bates et al., 2015). The dependent variable was mean ERP amplitude in the significant cluster. Fixed effects included all main effects and interactions between stimulus memory status (old, new), outcome condition (zero outcome, gain, loss), and trial number. Note that trial number was included as a fixed covariate to model the time course of ERP responses across the test session, rather than as a random factor to capture trial-level noise. Participant ID was included as a random intercept. To maintain a 5% Type I error rate, all identifiable random slopes were included (Barr et al., 2013; Schielzeth & Forstmeier, 2009), specifically for stimulus memory status, outcome condition, trial number (dummy-coded and centered), and all possible two-way interactions within participant ID. Before fitting the models, trial number was z-transformed (mean-centered and scaled) to provide a valid reference level for this predictor. The correlation parameters between random intercept and slopes were initially included, but were removed if their values were essentially one, making them unidentifiable (Matuschek et al., 2017).

We tested overall significance of fixed effects using a full-null model comparison (Forstmeier & Schielzeth, 2011), and significance of individual predictors using Satterthwaite approximation (Luke, 2017) via the function *lmer* from the *lmerTest* package (version 3.1–3; Kuznetsova et al., 2017). In cases where the three-way interaction was not significant, a reduced model with only the main effects and the two-way interactions was fitted to better estimate the two-way interaction effects.

For all reported effects, we provide unstandardized beta coefficients (β), standard errors (SE), *p*-values from Satterthwaite’s method and confidence intervals. Assumptions of normally distributed residuals were assessed using QQ-plots, and residuals were plotted against fitted values to check homoscedasticity Quinn & Keough, 2002). Model stability at the level of estimated coefficients and standard deviation was assessed by excluding one level of the random effect (i.e., participant ID) at a time and refitting the model (Nieuwenhuis et al., 2012), using a function kindly provided by Roger Mundry (University of Göttingen). Confidence intervals for the model estimates were obtained by parametric bootstrapping using the *bootMer* function from the *lme4* package, with 1000 bootstraps.^1^ The absence of collinearity issues was verified by calculating the Variance Inflation Factors (VIF) for a model without interaction terms, using the *vif* function from the *car* package (version 3.0–11; Fox & Weisberg, 2018).

To interpret significant fixed-effect interactions, we computed estimated marginal means and model-implied contrasts from the fitted LMMs using the *emmeans* package (version 1.11.2–8; Lenth, 2025). All simple effects were tested as linear contrasts of the fitted model (no model refitting), degrees of freedom were computed by using the Satterthwaite approximation (*lmerTest*), and *p*-value were multiplicity-adjusted by using Holm method (Holm, 1979).

#### Between-experiment analysis of stimulus memory status effect on ERP amplitudes

Following a preliminary analysis, we observed a marked disparity in the magnitude of old/new effect between the two experiments. To further explore this observation, an additional ERP analysis was conducted to examine potential differences in the temporal and spatial extent of the old/new effect across the two experiments.

To this aim, a mass univariate analysis was used to determine any interaction effect of stimulus memory status and experiment (i.e., character- or font-association group) on ERP amplitudes in the same P300/LPC ROI derived from the literature (300–600 ms, including electrodes Pz, POz, P1, P2, P3, P4, PO3, and PO4). We implemented a two-way ANOVA at each electrode–time point combination within this ROI, using *memory status* (old, new), *experiment* (1, 2), and their interaction as factors. Cluster-based permutation testing was used to correct for multiple comparisons, and only interaction clusters were retained for interpretation. This allowed us to test whether the spatial or temporal distribution of old/new effects differed significantly between experiments.

## Results

### Learning phase performance

To contextualize recognition performance in the test session, we first report participants’ learning success from the preceding session. As detailed in Grassi et al. (2023), participants demonstrated successful acquisition of the stimulus–outcome associations: during the final quarter of the learning session, average accuracy reached 97.22% (*SD* = 7.57%) in the character-association group and 91.16% (*SD* = 15.80%) in the font-association group. This indicates that participants reliably associated the visual stimuli with their respective outcome categories, validating subsequent analyses of test-phase performance as reflecting the influence of previously learned motivational relevance.

### Effects of stimulus memory status and associated outcome on behavior

Table S1 in the supplementary material shows a summary of the standard recognition memory performance measures by outcome condition for the two experiments.

The Bayesian regression models revealed overall very high performance in the old/new decision task, with the average probability of correctly assigning the memory status category exceeding 95% in both experiments.

**Fig. 2:**
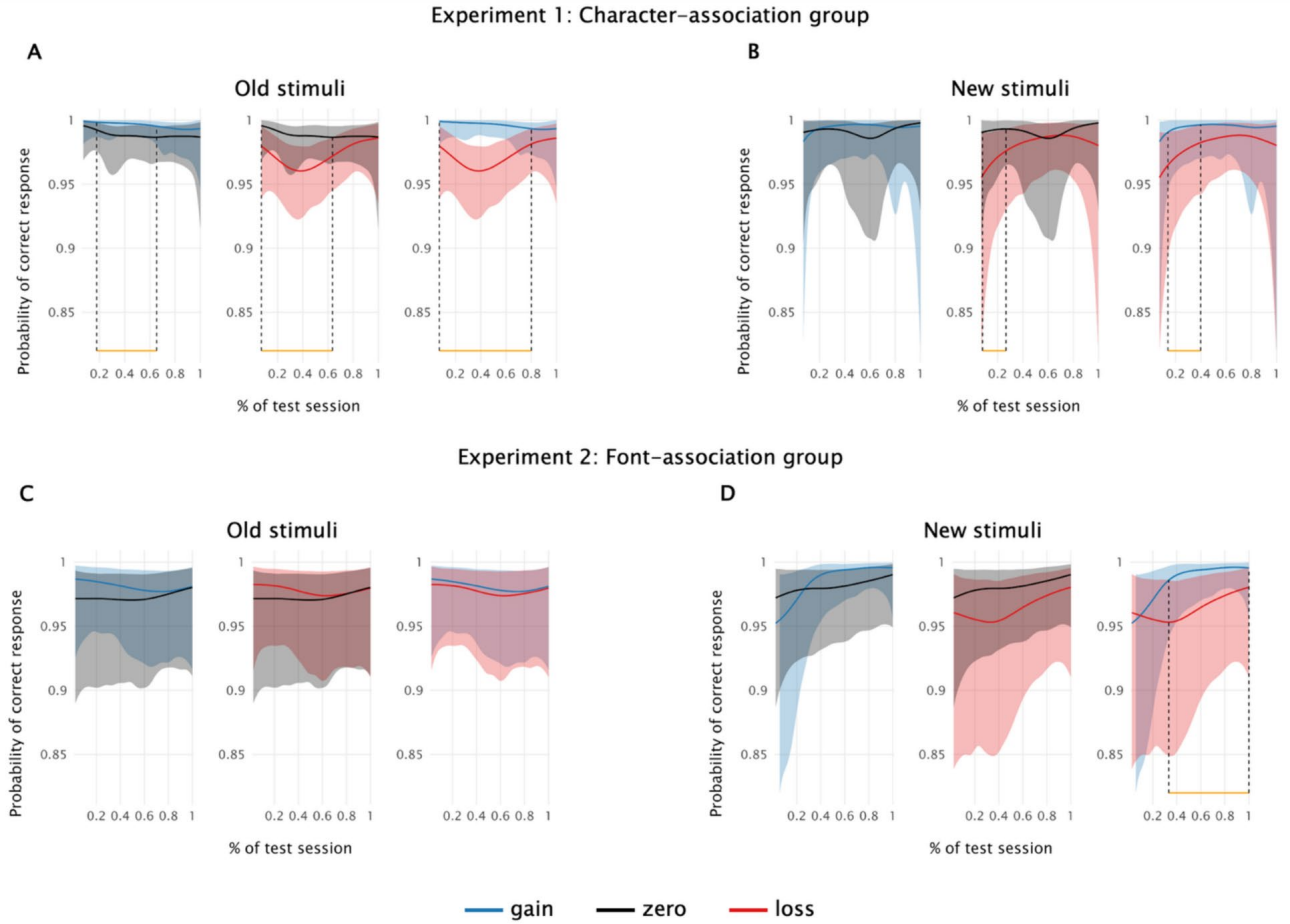
Mean probabilities to attribute memory status category correctly per outcome condition across the test session of Experiment 1 (top) and Experiment 2 (bottom), for old (**A, C**) and new (**B, D**) stimuli separately. Each subpanel shows the comparison between two outcome conditions. Differences between outcome conditions outside the 99% confidence bands are indicated by the orange segments along the x-axis

In the character-association group, significant differences in participant responses to stimuli associated with different outcome conditions were observed for both old and new stimuli (Figs. 2A, B). For the old stimuli, the probability of a correct response was higher for gain-associated stimuli compared to both loss-associated and zero outcome stimuli, particularly during the first half of the test session. Specifically, the probability of correct responses for gain-associated stimuli exceeded that for loss-associated stimuli from 7 to 80% of the test session duration, and that for zero outcome stimuli from 18 to 65% of the test session duration. Similarly, zero outcome stimuli showed a higher probability of correct response compared to loss-associated stimuli during 7% to 64% of the session duration. For new stimuli, significant differences were observed during the first part of the session: gain- and zero outcome stimuli each outperformed loss-associated stimuli during 14% and 40%, and 8% to 27% of the session duration, respectively).

In the font-association group, significant differences in participant responses were observed only for the new stimuli, with gain-associated stimuli showing higher probability of correct responses compared with loss-associated stimuli during 33% and 100% of the test session duration (Figs. 2C, D).

These findings suggest that motivational relevance, particularly gain associations, enhanced recognition accuracy—most clearly in the character-association group and primarily for old stimuli. This pattern supports the hypothesis that positive value associations facilitate memory performance, especially when aligned with more salient perceptual features.

### Effects of stimulus memory status on ERP amplitudes

**Fig. 3:**
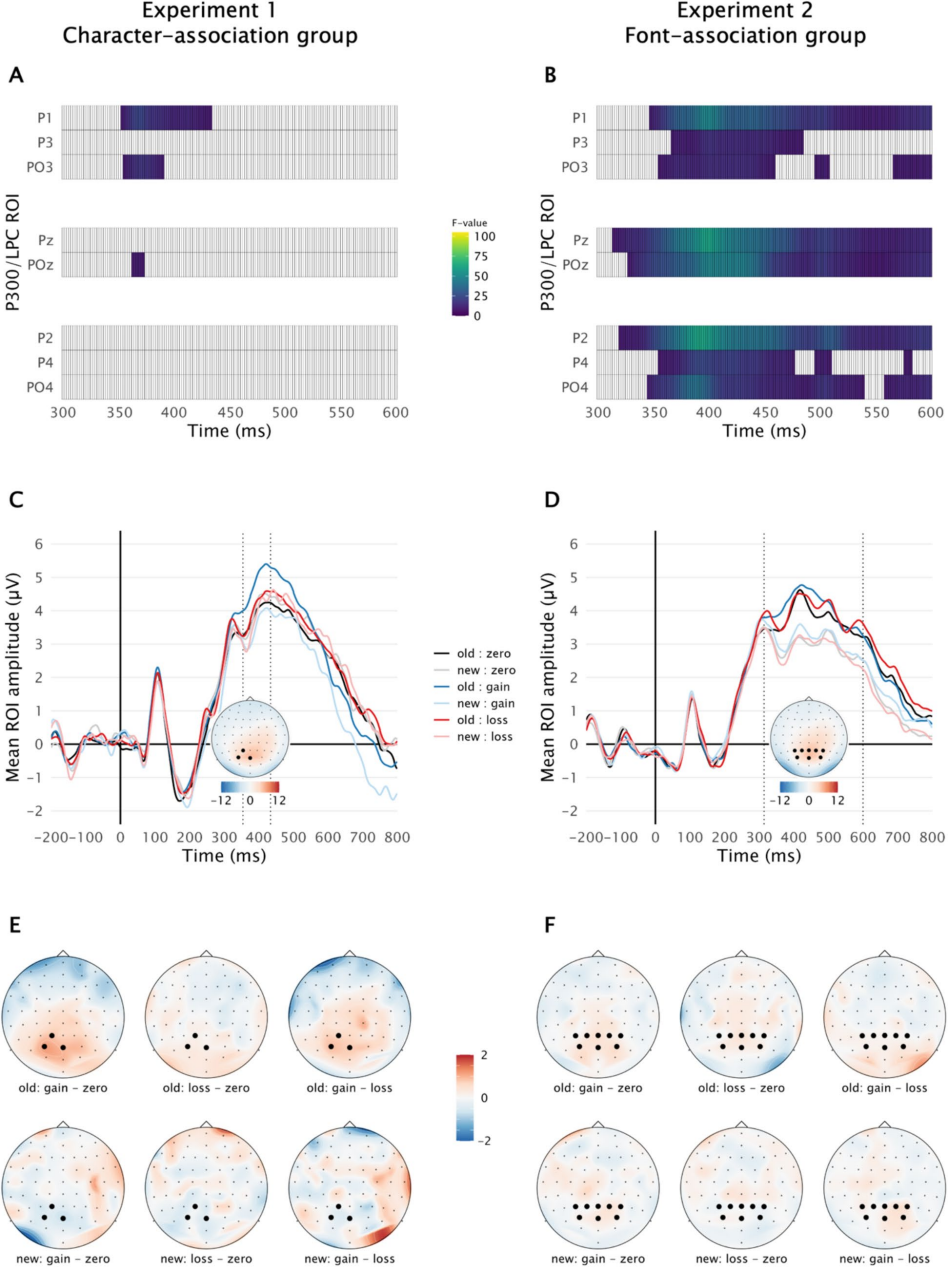
P300/LPC modulations for the character-association group (left column) and the font-association group (right column). (**A, B**) Significant clusters of old/new effects in the predefined spatiotemporal ROI. (**C, D**) Grand-averaged ERPs at the significant cluster’s electrodes, with corresponding scalp topographies of grand-averaged ERP across all conditions. Dotted lines represent the temporal extent of the significant cluster. (**E, F**) Scalp topographies averaged across the time window of the significant cluster, contrasted for zero, gain and loss outcomes, and old and new stimuli

In the character-association group, the mass univariate analysis of the P300/LPC spatiotemporal ROI revealed a significant effect of stimulus memory status in a cluster comprising electrodes P1, PO3, and POz, between 354 and 434 ms (*cluster mass* = 893.22, *p* =.008; see Fig. 3A). The cluster peaked spatially at electrode P1 and temporally at 369 ms. In the font-association group, the analysis revealed a significant effect of stimulus memory status in a cluster including electrodes P1, P3, PO3, Pz, POz, P2, P4, and PO4, between 314 and 600 ms (*cluster mass* = 19,179.26, *p* <.001; Fig. 3B). This cluster peaked spatially at electrode P2 and temporally at 398 ms. In both experimental groups, the average amplitudes extracted from the significant clusters in the P300/LPC ROI exhibited the typical waveform and topography associated with this component (Fig. 3C, E and D, F, respectively).

The ERP findings confirm robust old/new effects in the P300/LPC time window for both experiments, with a broader and more sustained cluster in the font-association group. This indicates that stimulus memory status reliably modulated neural activity, though the spatial and temporal extent of this effect differed by encoding dimension.

### Temporal development of stimulus memory status and associated outcome effects

The LMMs used to test the interaction effect of stimulus memory status, outcome condition, and trial number on the mean amplitudes extracted from the significant clusters in the P300/LPC ROI revealed no violations of model assumptions and demonstrated acceptable model stability across both experimental groups. A summary of these LMM results is presented in Tables 2, 3, 4. For both groups, the models were fitted without including the correlation of random intercepts and slopes due to highly correlated random effects (≥.9), which resulted in only a minor decrease in model fit (Table 5).

**Table 2:** Summary of the LMMs regarding the effect of stimulus memory status, outcome condition, and trial on average P300/LPC amplitudes, using (new) as reference level for stimulus memory status and (zero outcome) as reference level for outcome condition

	Experiment 1: Character-Association Group						Experiment 2: Font-Association Group					
*Predictors*	*R2*	*Estimate*	*std. Error*	*95% CI*	*t value*	*p*	*R2*	*Estimate*	*std. Error*	*95% CI*	*t value*	*p*
	.010						.009					
Intercept		4.18	0.81	2.58 5.64		^(d)^		2.95	0.34	2.27 3.64		^(d)^
outcome (gain) ^a^		− 0.79	0.88	− 2.46 0.86	− 0.90	.367		0.17	0.17	− 0.12 0.48	1.02	.309
outcome (loss) ^a^		− 1.37	0.89	− 2.95 0.43	− 1.55	.122		− 0.07	0.16	− 0.37 0.24	− 0.42	.675
memory status (old) ^b^		− 0.22	0.64	− 1.41 1.04	− 0.35	.728		0.79	0.20	0.40 1.18	3.88	<.001
trial ^c^		0.24	0.70	− 1.14 1.60	0.34	.732		− 0.01	0.13	− 0.25 0.24	− 0.05	.964
outcome (gain) ^a^ * memory status (old) ^b^		1.80	0.91	0.07 3.52	1.98	.047		0.15	0.21	− 0.30 0.57	0.70	.482
outcome (loss) ^a^ * memory status (old) ^b^		1.54	0.90	−0.32 3.09	1.70	.089		0.26	0.21	− 0.19 0.67	1.25	.211
outcome (gain) ^a^ * trial ^c^		− 0.39	0.97	− 2.30 1.47	− 0.40	.690		0.02	0.15	− 0.29 0.30	0.10	.919
outcome (loss) ^a^ * trial ^c^		− 1.26	0.98	− 3.02 0.68	− 1.29	.198		− 0.07	0.16	− 0.39 0.25	−0.41	.684
memory status (old) ^b^ * trial ^c^		− 0.48	0.72	− 1.87 0.97	− 0.67	.501		0.37	0.16	0.06 0.69	2.39	.018
outcome (gain) ^a^ * memory status (old) ^b^ * trial ^c^		0.20	0.99	− 1.81 2.07	0.20	.841		− 0.51	0.21	− 0.91 − 0.08	− 2.41	.016
outcome (loss) ^a^ * memory status (old) ^b^ * trial ^c^		1.33	1.00	− 0.61 3.22	1.34	.181		− 0.21	0.21	− 0.65 0.20	− 1.00	.317

^a^ Comparison with the reference level (zero outcome)

^b^ Comparison with the reference level (new)

^c^ z-transformed to mean of zero and a standard deviation of one

^d^ Not shown because of being of very limited interpretability

CI = confidence interval

**Table 3:** Summary of the LMMs regarding the effect of stimulus memory status, outcome condition, and trial on average P300/LPC amplitudes, using (new) as reference level for stimulus memory status and (loss) as reference level for outcome condition

	Experiment 1: Character-Association Group						Experiment 2: Font-Association Group					
*Predictors*	*R2*	*Estimate*	*std. Error*	*95% CI*	*t value*	*p*	*R2*	*Estimate*	*std. Error*	*95% CI*	*t value*	*p*
	.010						.009					
Intercept		2.81	0.80	1.31 4.34		^(d)^		2.89	0.34	2.21 3.51		^(d)^
outcome (gain) ^a^		0.57	0.88	− 1.11 2.31	0.65	.515		0.23	0.17	− 0.13 0.56	1.37	.174
outcome (zero) ^a^		1.36	0.89	− 0.44 3.19	1.53	.127		0.06	0.15	− 0.23 0.35	0.43	.669
memory status (old) ^b^		1.31	0.64	0.10 2.62	2.05	.040		1.05	0.20	0.65 1.48	5.14	<.001
trial ^c^		− 1.02	0.69	− 2.49 0.30	−1.48	.140		− 0.08	0.13	− 0.34 0.16	− 0.62	.537
outcome (gain) ^a^ * memory status (old) ^b^		0.27	0.90	− 1.56 1.99	0.30	.767		− 0.12	0.21	− 0.56 0.31	− 0.56	.573
outcome (zero) ^a^ * memory status (old) ^b^		− 1.53	0.91	− 3.43 0.30	−1.68	.092		− 0.26	0.22	− 0.72 0.17	− 1.20	.234
outcome (gain) ^a^ * trial ^c^		0.87	0.96	− 0.98 2.82	0.90	.369		0.09	0.15	− 0.19 0.40	0.63	.527
outcome (zero) ^a^ * trial ^c^		1.25	0.98	− 0.61 3.26	1.28	.202		0.07	0.16	− 0.23 0.41	0.46	.650
memory status (old) ^b^ * trial ^c^		0.84	0.70	−0.45 2.34	1.20	.231		0.16	0.15	−0.15 0.46	1.05	.295
outcome (gain) ^a^ * memory status (old) ^b^ * trial ^c^		− 1.12	0.98	− 3.13 0.77	− 1.15	.252		− 0.30	0.21	− 0.72 0.12	−1.42	.157
outcome (zero) ^a^ * memory status (old) ^b^ * trial ^c^		− 1.32	1.00	− 3.29 0.61	− 1.32	.186		0.21	0.21	− 0.24 0.59	0.98	.325

^a^ Comparison with the reference level (loss)

^b^ Comparison with the reference level (new)

^c^ z-transformed to mean of zero and a standard deviation of one

^d^ Not shown because of being of very limited interpretability

**Table 4:** Summary of the reduced LMMs regarding the effect of stimulus memory status, outcome condition, and trial on average P300/LPC amplitudes in Experiment 1, using (new) as reference level for stimulus memory status and either (zero outcome) or (loss) as reference level for outcome condition

	Experiment 1: Character-Association Group					
*Predictors*	*R2*	*Estimate*	*std. Error*	*95% CI*	*t value*	*p*
	.006					
Outcome Condition Reference Level: Zero Outcome						
intercept		3.77	0.66	2.61 5.01		^(c)^
outcome (gain)		− 0.64	0.37	− 1.32 0.07	−1.73	.084
outcome (loss)		− 0.29	0.37	− 0.97 0.38	−0.79	.432
memory status (old) ^a^		0.19	0.44	− 0.65 0.99	0.42	.676
trial ^b^		− 0.25	0.42	− 1.02 0.57	−0.61	.543
outcome (gain) * memory status (old) ^a^		1.64	0.44	0.79 2.41	3.72	<.001
outcome (loss) * memory status (old) ^a^		0.47	0.42	− 0.29 1.23	1.10	.272
outcome (gain) * trial ^b^		− 0.20	0.19	− 0.56 0.18	−1.05	.301
outcome (loss) * trial ^b^		0.02	0.19	− 0.32 0.39	0.12	.903
memory status (old) ^a^ * trial ^b^		0.03	0.41	− 0.76 0.83	0.07	.941
Outcome Condition Reference Level: Loss						
Intercept		3.47	0.65	2.18 4.79		^(c)^
outcome (gain)		− 0.34	0.36	− 1.09 0.37	− 0.95	.343
outcome (zero)		0.29	0.37	− 0.46 1.03	0.78	.441
memory status (old) ^a^		0.66	0.44	− 0.22 1.55	1.49	.138
trial ^b^		− 0.24	0.42	− 1.04 0.59	− 0.57	.570
outcome (gain) * memory status (old) ^a^		1.17	0.43	0.34 2.04	2.72	.007
outcome (zero) * memory status (old) ^a^		− 0.47	0.43	− 1.31 0.39	− 1.09	.277
outcome (gain) * trial ^b^		− 0.22	0.19	− 0.60 0.16	− 1.15	.258
outcome (zero) * trial ^a^		− 0.02	0.18	− 0.38 0.31	− 0.12	.902
memory status (old) ^a^ * trial ^b^		0.03	0.41	− 0.81 0.82	0.08	.937

^a^ Comparison with the reference level (new)

^b^ z-transformed to mean of zero and a standard deviation of one

^c^ not shown because of being of very limited interpretability

**Table 5:** Comparison of the log-likelihoods of the models fitted including or lacking the correlation of random intercepts and slopes

	Including Correlation Parameters		Lacking Correlation Parameters	
*Model*	Log-likelihood	df	Log-likelihood	df
Experiment 1	− 28,889.44	68	− 28,910.33	23
Experiment 2	− 40,832.70	68	− 40,853.68	23

**Fig. 4:**
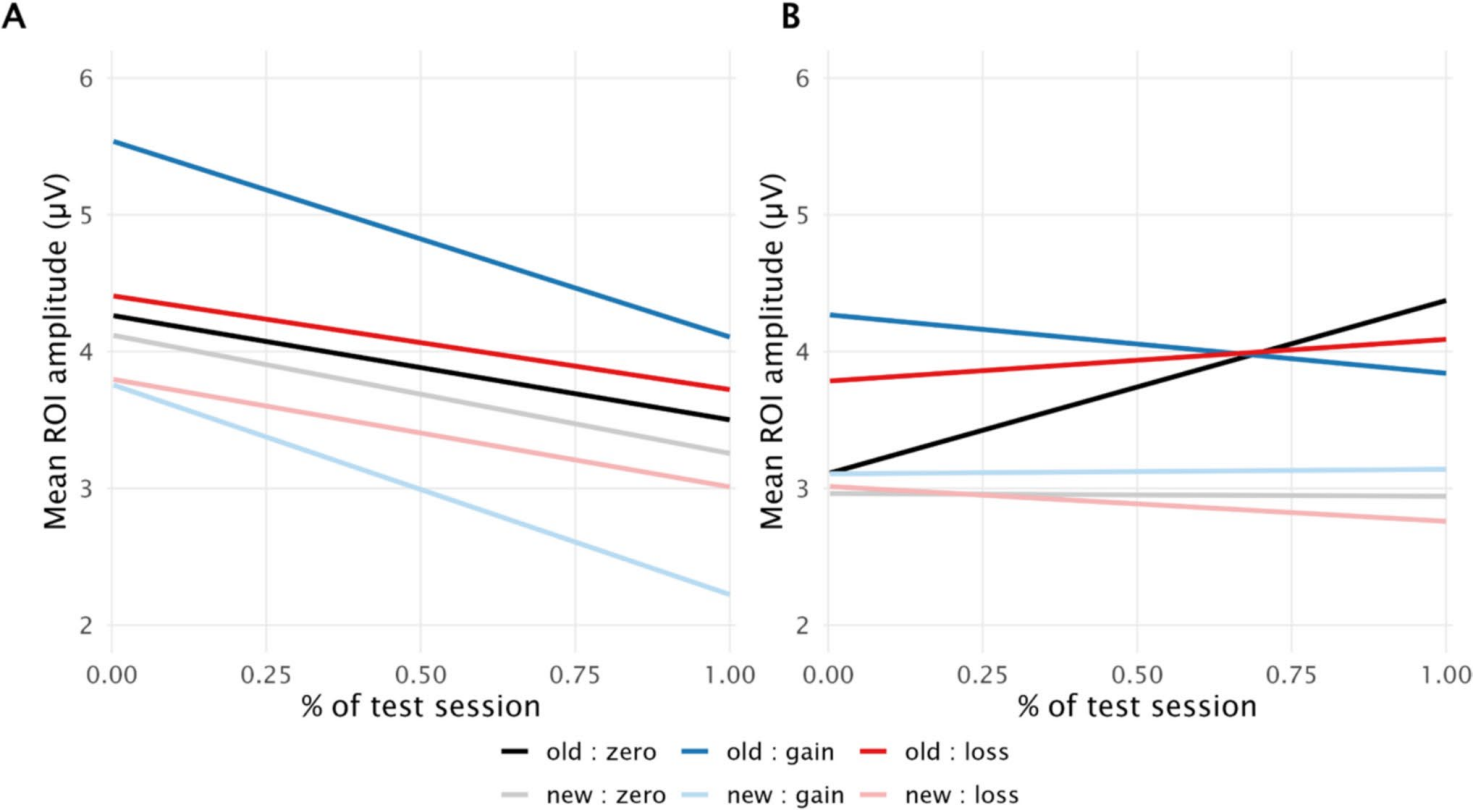
Average ERP amplitudes at the significant cluster electrodes, over the test session, contrasted for zero, gain and loss outcomes, and old and new stimuli. (**A**) Experiment 1: character-association group. (**B**) Experiment 2: font-association group

For the character-association group, the model did not reveal a significant three-way interaction. However, there was a significant interaction effect between stimulus memory status and associated outcome (likelihood ratio test for full-null model comparison: χ^2^ = 36.70, *df* = 11, *p* <.001). Simple outcome effects were calculated within each level of memory status condition. Within the old stimuli, mean amplitudes in the P300/LPC cluster were significantly enhanced for gain-associated stimuli compared with loss-associated (*β* = 0.83, *SE* = 0.26, *p* =.004, 95% confidence interval [CI] [0.21, 1.44]) and zero outcome stimuli (*β* = 1.00, *SE* = 0.24, *p* =.001, 95% CI [0.43, 1.58]; Fig. 4A) while no difference was observed for new stimuli (gain vs. loss: *β* = − 0.35, *SE* = 0.38, *p* =.356, 95% CI [− 1.20, 0.50]; gain vs. zero: *β* = −0.64, *SE* = 0.37, *p* =.169, 95% CI [− 1.46, 0.19]). For the font-association group, the model revealed a significant three-way interaction between stimulus memory status, outcome condition, and trial number (likelihood ratio test for full-null model comparison: *χ*^*2*^ = 39.14, *df* = 11, *p* <.001). Contrasted slopes of trial between outcome levels were calculated within each memory status level. Within the old stimuli, zero outcome stimuli showed a significantly steeper slope of trial compared to gain-associated stimuli (*β* = 0.49, *SE* = 0.15, *p* =.003, 95% CI [0.13 0.85]) while no difference was observed between zero outcome and loss-associated stimuli (*β* = 0.28, *SE* = 0.17, *p* =.194, 95% CI [− 0.13 0.68]) or between gain- and loss-associated stimuli (*β* = − 0.21, *SE* = 0.17, *p* =.201, 95% CI [− 0.62 0.19]). Within the new stimuli, no significant difference was observed between any level of outcome condition (zero vs. gain: *β* = − 0.02, *SE* = 0.15, *p* = 1.000, 95% CI [− 0.37 0.34]; zero vs. loss: *β* = 0.07, *SE* = 0.16, *p* = 1.000, 95% CI [− 0.33 0.47]; gain vs. loss: *β* = 0.08, *SE* = 0.16, *p* = 1.000, 95% CI [−0.32 0.48]). As can be seen in Fig. 4B, at the beginning of the test session, within the old stimuli, mean amplitudes in the P300/LPC cluster were enhanced for gain-associated stimuli compared to zero outcome stimuli. In contrast, within the new stimuli, no differences between outcome conditions were observed. However, towards the end of the session, the amplitude difference between the gain and zero outcome conditions within the old stimuli decreased, and all old stimuli showed enhanced amplitudes compared to the new stimuli.

These results indicate that gain-associated stimuli elicited the strongest P300/LPC enhancements, particularly for old items and early in the test session. While the character-association group showed a stable relevance-by-memory status interaction, the font-association group exhibited a dynamic pattern: initial gain-related enhancements gave way to a general old/new effect over time. This suggests shifting recognition strategies as the session progressed.

### Between-experiment differences of stimulus memory status effects on ERP amplitudes

**Fig. 5:**
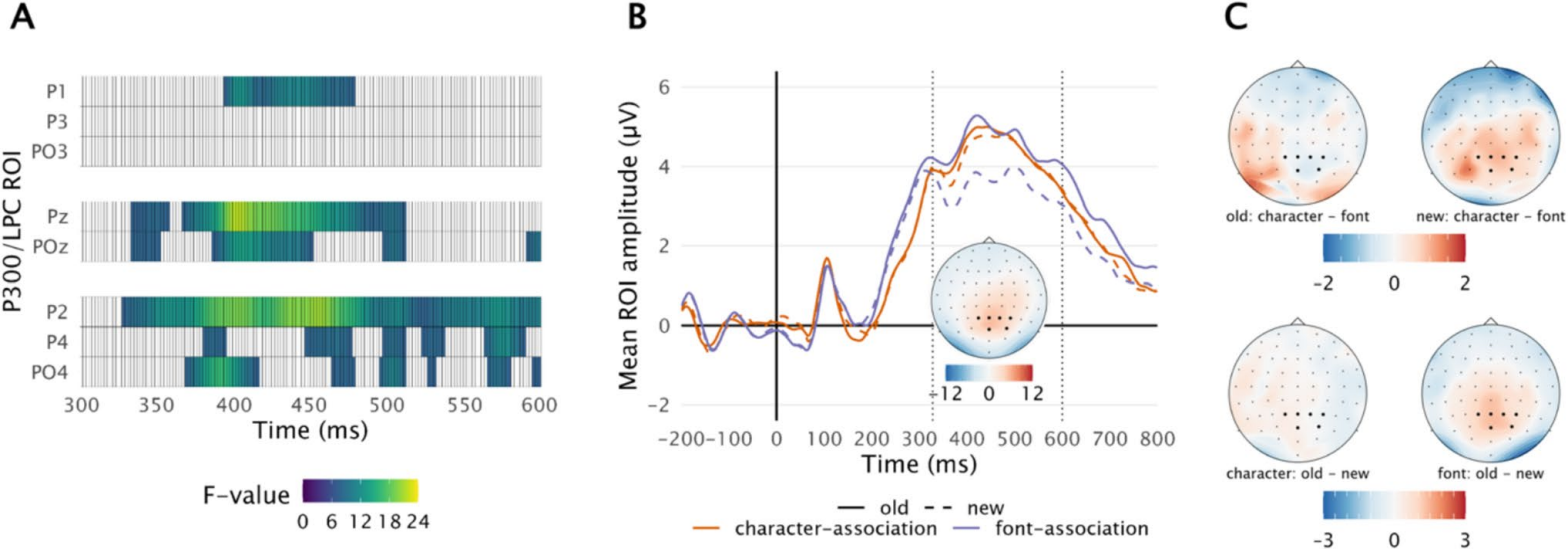
P300/LPC modulations by stimulus memory status and experiment. (**A**) Significant cluster of interaction effects of stimulus memory status and experiment in the predefined spatiotemporal ROI. (**B**) Grand-averaged ERPs at the significant cluster’s electrodes, with corresponding scalp topography of grand-averaged ERP across all conditions. Dotted lines represent the temporal extent of the significant cluster. (**C**) Scalp topographies averaged across the time window of the significant cluster, contrasted for the two experimental groups, for old and new stimuli separately (top) and for old and new stimuli, for the two experiments separately (bottom)

Figure 5 shows the results of the mass univariate analysis testing the effects of stimulus memory status, experiment, and their interaction on the P300/LPC spatiotemporal ROI. The analysis revealed a significant interaction effect of stimulus memory status and experiment in a cluster comprising electrodes P1, Pz, POz, P2, P4, and PO4, between 328 and 600 ms (*p* <.001), with a spatial peak located at electrode Pz, and a temporal peak at 402 ms.

The average amplitudes extracted from the significant cluster exhibited the typical waveform and topography associated with the P300/LPC component (Figs. 5B, C). Importantly, this interaction effect indicates that the old/new ERP difference was more broadly distributed and temporally sustained in the font-association group (Experiment 2) compared with the character-association group (Experiment 1). Specifically, in Experiment 2, the old/new effect extended throughout the full P300/LPC time window and across a wider set of posterior electrodes, whereas in Experiment 1, it was more temporally circumscribed and spatially restricted. This pattern suggests that the font-association manipulation in Experiment 2 may have elicited stronger or more prolonged memory-related neural responses, possibly reflecting greater difficulty or ambiguity in the recognition process when font rather than character identity was the relevant learning dimension.

## Discussion

Building on the behavioral and ERP results outlined above, we now discuss their implications for memory enhancement, value-based prioritization, and the role of perceptual features in recognition processes.

The present study aimed to elucidate the interplay between motivational relevance and stimulus memory status in visual word processing, as reflected by P300/LPC amplitude modulations. While our stimuli consisted of meaningless consonant strings, these engage key sublexical mechanisms of the visual word recognition system, such as early orthographic and perceptual processing stages (Dehaene et al., 2005; Grainger & Holcomb, 2009), which are foundational to visual word processing. To achieve our aim, we conducted two parallel experiments that orthogonally manipulated motivational relevance and stimulus memory status within an old/new decision task. To complement the electrophysiological data, we also assessed participants’ performance in the old/new decision task. Behavioral data from both experiments revealed high accuracy, with participants categorizing more than 95% of stimuli correctly on average. Notably, gain-associated stimuli exhibited the highest accuracy, suggesting that motivational relevance facilitated the recognition and categorization of old stimuli. In Experiment 1 (character-association group), gain-associated old stimuli were more accurately identified than loss-associated and zero outcome stimuli, with a similar trend observed in Experiment 2 (font-association group). These behavioral findings are consistent with the ERP results discussed below, which illustrate how motivational relevance and stimulus old/new status modulated P300/LPC amplitudes.

Our primary hypothesis was that stimuli previously associated with monetary gain would elicit enhanced P300/LPC amplitudes, particularly when they were old. This prediction was grounded in prior research indicating that gain-associated symbolic stimuli elicit stronger memory-related ERP responses than neutral or loss-associated stimuli (Grassi et al., 2023; Rossi et al., 2017). Although exploratory analyses included all three outcome conditions, the strongest a priori prediction focused on the neural prioritization of gain-associated stimuli.

Experiment 1 (character-association group) showed clear support for this prediction, as we observed a significant interaction effect of associated relevance and stimulus memory status on these neural measures. Specifically, old stimuli associated with monetary gain elicited increased P300/LPC amplitudes compared to other conditions, confirming our hypothesis that motivationally relevant stimuli receive preferential neural processing. This neural prioritization paralleled the behavioral advantage observed for gain-associated stimuli, supporting a functional link between P300/LPC modulations and improved memory performance. These findings reinforce the idea that reward-associated stimuli benefit from enhanced discrimination and recollection, consistent with previous work (Hammerschmidt et al., 2018; Rossi et al., 2017). Importantly, in this experiment, participants were required to associate monetary outcomes with the characters of the stimuli during the learning session, disregarding the font. Participants had minimal incentive to memorize the font during the learning session and could base their recognition primarily on the characters during the test session, possibly also due to the higher functional relevance of characters in everyday word processing. Consequently, one might have anticipated that any relevance effect linked to the characters of old stimuli would generalize to new stimuli differing only in font. However, our results demonstrated that the increased P300/LPC amplitudes due to gain-association were confined to old stimuli. This suggests that the positive monetary association enhanced the memory of specific stimulus features—the character-font pair encountered during learning—thereby facilitating their discrimination and recollection during the test session. Importantly, ERP data from the encoding phase of this paradigm, published in a separate study (Grassi et al., 2023), provide complementary evidence for early and sustained processing advantages of gain-associated stimuli. These encoding-related findings suggest that the observed retrieval effects are likely grounded in differential attentional engagement and memory formation during the learning phase.

Despite the clear effect in Experiment 1, we did not observe a statistically significant three-way interaction involving associated relevance, stimulus old/new status, and trial number. Although visual inspection suggested a trend toward a decreasing amplitude difference between gain-associated old stimuli and other conditions, we refrain from interpreting this as an observed effect. It remains speculative and warrants further investigation. Two nonmutually exclusive mechanisms could potentially explain this pattern if it were replicated: First, the absence of monetary outcomes or feedback during the test session may have led to the extinction of the relevance effect, mirroring a gradual reduction in the behavioral advantage for gain-associated stimuli. Second, repeated exposure to new stimuli during the test session likely increased their familiarity, thereby decreasing their distinctiveness relative to old stimuli. As novel stimuli became more familiar, the old/new ERP effect might progressively weaken. However, as this pattern was not statistically confirmed, these explanations remain tentative.

Experiment 2 (font-association group) allowed us to assess whether the same pattern of effects would emerge under a different learning manipulation, with participants focusing on font rather than character associations. A key difference in Experiment 2 was the emergence of a three-way interaction between associated relevance, stimulus memory status, and trials. At the outset of the test session, old stimuli associated with monetary gain again elicited increased P300/LPC amplitudes, mirroring the findings of Experiment 1. As the test session progressed, however, the amplitude differences between old and new stimuli appeared to become less dependent on motivational relevance, suggesting a shift from early relevance-based prioritization toward a more general memory-based processing strategy. This pattern aligns with previous electrophysiological evidence (Bayer et al., 2019; Fritsch & Kuchinke, 2013; Kuchinke et al., 2015; Kulke et al., 2019; Rossi et al., 2017) and supports the notion that motivationally relevant stimuli receive prioritized processing early on, but this effect diminishes as recognition strategies adapt over time. In Experiment 2, old and new stimuli differed in their characters but shared the same font. As previously discussed, participants may have naturally prioritized characters, given their greater salience in everyday word processing. This bias may have been particularly pronounced during the test session, where participants had no specific instruction or feedback to focus on font. At the beginning of the test session, the enhanced P300/LPC amplitudes for gain-associated old stimuli likely reflected an initial boost in recollection for specific stimulus features, consistent with the behavioral advantage observed in Experiment 1. However, as the session progressed and participants relied on their own recognition strategies, their focus may have shifted toward characters as a more intuitive cue for distinguishing old from new stimuli. By the end of the session, the relevance effect had largely dissipated, and P300/LPC modulations were predominantly driven by the memory status (old versus new) of the character set, independent of the font’s consistency across stimuli.

Across both experiments, P300/LPC amplitude modulations were selectively enhanced for stimuli associated with positive motivational relevance (monetary gain), with no such effect observed for loss-associated stimuli. This asymmetry is consistent with our gain-focused hypothesis and aligns with behavioral findings from previous studies (Ferré, 2003; Grassi et al., 2023, 2024; Rossi et al., 2017; Schacht et al., 2012), where positive symbolic stimuli were recognized better and produced faster reaction times than both neutral and negative stimuli. Our ERP findings complement this behavioral pattern, suggesting that the observed P300/LPC enhancements for gain-associated stimuli may underlie their superior recognition. In contrast, some electrophysiological studies have shown that the old/new P300/LPC modulations are particularly pronounced for negative stimuli in the context of real words with intrinsic emotional content (Dietrich et al., 2001; Rossi et al., 2017; Weymar et al., 2013). This discrepancy may be explained by differences in salience and representational richness between symbolic and semantic stimuli. In the absence of semantic content, positively valenced associations may more reliably engage attention and recollection-related processes, possibly due to their alignment with dopaminergic reward systems and motivational approach tendencies (Bayer et al., 2019; Grassi et al., 2023; Schacht et al., 2012). In contrast, loss associations based on symbolic stimuli, such as monetary penalties in a lab setting, may lack the emotional immediacy or biological relevance needed to activate avoidance-related memory systems to a comparable degree. Unlike negative words or threatening images, abstract loss associations may not evoke sufficient affective engagement to elicit sustained ERP effects during recognition.

Even outside the domain of memory processing, the literature concerning specific P300/LPC modulations for symbolic stimuli of positive or negative associated relevance is somewhat mixed (Fritsch & Kuchinke, 2013; Grassi et al., 2023; Montoya et al., 1996; Rossi et al., 2017). For instance, Schacht et al. (2012) proposed that P300/LPC modulations reflect a valence-unspecific relevance evaluation process, with comparable effects for both positive and negative stimuli. Further investigations are needed to elucidate potential differences in the effects of positive and negative associated relevance on the stimulus processing stages underlying P300/LPC amplitudes.

Another key finding of the present study is the differential robustness of the old/new ERP effects across the two experiments. The character-association group (Experiment 1) showed stronger and more temporally sustained P300/LPC modulations than the font-association group (Experiment 2). We interpret this as evidence that the perceptual dimension used to encode motivational relevance plays a critical role in recognition memory. Characters are highly salient and functionally central in visual word processing, which likely made them easier to encode and retrieve. In contrast, font type is a less prominent and more variable feature that is not typically prioritized during reading. As such, associations formed with font may have been less robust, especially once reinforcement was removed. This may have contributed to the reduced and less consistent ERP effects observed in Experiment 2.

Although we refer to “character prioritization” throughout the paper, we wish to clarify that this inference is based on indirect evidence. Specifically, stronger and more temporally stable old/new ERP effects in Experiment 1 (where characters carried the associations) relative to Experiment 2 (where fonts were relevant) suggest that participants may have spontaneously relied more heavily on character-based familiarity during recognition. In Experiment 2, this interpretation is further supported by the observation that P300/LPC modulations increasingly reflected stimulus memory status rather than motivational relevance as the test session progressed—consistent with a shift away from font-based retrieval strategies. However, as these conclusions are drawn from differences in ERP patterns across experiments, rather than from a direct manipulation of feature prioritization, they should be interpreted with caution.

## Limitations and future directions

Despite the robustness of our findings, several limitations should be considered. First, the overall accuracy in the old/new recognition task was very high across participants (> 95%), leaving few incorrect responses (i.e., false alarms or misses) for analysis. This limited our ability to examine how motivational relevance may differentially affect correct versus incorrect memory decisions—a distinction that is often central in the study of recognition memory. Moreover, because the task required only binary judgments (old vs. new), we were unable to distinguish between qualitatively different memory processes, such as recollection (retrieving specific contextual details) versus familiarity (a general sense of knowing). These two processes are thought to rely on distinct neural mechanisms and may be differentially modulated by motivational factors. In this context, an exploratory analysis of frontal electrode activity (see Supplementary Material S2) revealed a small left-lateralized interaction between experiment and memory status in the 300–500 ms range, consistent with possible familiarity-related processes. However, given its exploratory nature and limited interpretability, this finding should be considered tentative. Future studies could address this by using more challenging recognition paradigms that introduce greater uncertainty (e.g., via more similar foils) or by employing graded response formats, such as “remember,” “know,” or “guess” judgments. Such paradigms would allow for a more fine-grained characterization of how motivational associations influence memory processes, and whether their effects are driven more by enhanced familiarity, recollection, or both. Finally, our exclusive use of symbolic, nonsemantic stimuli provides a strong test of low-level associative learning mechanisms, but also limits direct generalization to ecologically valid reading or memory situations involving meaningful content.

## Conclusion

This study investigated how motivational relevance and stimulus memory status interact to shape recognition memory for nonsemantic visual stimuli. Across two experiments, stimuli associated with monetary gain elicited enhanced P300/LPC amplitudes and improved recognition accuracy—effects that were most pronounced for old stimuli and aligned with prior evidence linking reward associations to memory enhancement (Grassi et al., 2023; Rossi et al., 2017).

We also found that the perceptual dimension used to encode motivational relevance shaped the robustness of these effects. Associations tied to character identity, a salient and functionally central feature in word recognition (Dehaene et al., 2005; Grainger & Holcomb, 2009), produced more sustained ERP modulations than associations tied to font, suggesting that perceptual salience plays a key role in value-based memory prioritization.

Together, these findings extend previous research on emotional and motivational influences on memory (Citron, 2012; Schacht & Sommer, 2009), demonstrating that even in the absence of semantic content, learned motivational relevance can enhance both neural and behavioral markers of recognition memory.

## Supplementary Information

Below is the link to the electronic supplementary material.

Supplementary file1 (DOCX 301 kb)

## Data Availability

Stimulus material used in the experiment, as well as processed EEG and behavioral data is publicly available at the Open Science Framework and can be accessed at https://osf.io/grw9b/?view_only=0abef24b1d7442bf97007f4e3abbcb88. Raw EEG data are available upon request to the authors.

## Footnotes

1 Due to the different computation methods, the interpretation of Satterthwaite approximated p-values and bootstrapped CIs might differ.

## Code availability

Custom code used in the experimental procedure, data pre-processing and statistical testing is publicly available at the link provided above.

## Declarations

### Open Practices Statement

The study was not preregistered.
