# Supplementary material for: Meaningless but memorable: Reward associations boost recognition of abstract visual stimuli
Source: Cogn Affect Behav Neurosci. 2026 Mar 16;26(3):903–22. doi: 10.3758/s13415-025-01387-w (PMC13260039; doi:10.3758/s13415-025-01387-w)
Supplement: Supplementary file 1 — Supplementary file1 (DOCX 301 kb) [file 13415_2025_1387_MOESM1_ESM.docx]

Supplementary Material

S1. Recognition Memory Performance

**Table S1.** Mean and 95% confidence intervals of the recognition memory performance measures, by outcome condition.

|  | **Hits** | | **False Alarms** | | **d'** | | **Response Bias (c)** | |
| --- | --- | --- | --- | --- | --- | --- | --- | --- |
| *Outcome* | *Mean* | *95% CI* | *Mean* | *95% CI* | *Mean* | *95% CI* | *Mean* | *95% CI* |
| Experiment 1: Character-Association Group | | | | | | | | |
| zero | 117.67 | 116.40 118.93 | 0.62 | 0.22 1.03 | 4.19 | 3.92 4.47 | -0.07 | -0.15 0.00 |
| gain | 118.88 | 118.34 119.41 | 0.46 | 0.05 0.87 | 4.49 | 4.28 4.70 | -0.15 | -0.22 -0.08 |
| loss | 114.79 | 110.89 118.69 | 1.33 | 0.26 2.40 | 3.81 | 3.46 4.16 | -0.02 | -0.12 0.09 |
| Experiment 2: Font-Association Group | | | | | | | | |
| zero | 112.25 | 106.75 117.75 | 5.38 | 1.33 9.42 | 3.93 | 3.53 4.32 | 0.11 | -0.10 0.31 |
| gain | 114.04 | 109.59 118.49 | 3.71 | 0.75 6.66 | 4.15 | 3.77 4.53 | 0.05 | -0.13 0.24 |
| loss | 113.29 | 108.82 117.76 | 9.46 | -0.08 19.00 | 3.82 | 3.33 4.31 | 0.00 | -0.23 0.24 |

S2. Exploratory Analysis of Stimulus Memory Status Effects on ERP Amplitudes at Frontal Electrodes

An exploratory ERP analysis was conducted to examine potential differences in the temporal and spatial extent of the old/new effect at frontal electrode sites between the two experiments. The approach used was the same as in the analysis of between-experiment effects in the P300/LPC ROI, as described in the main text. This time the mass univariate analysis included electrodes AF3, Afz, AF4, F5, F3, F1, Fz, F2, F4, F6, FC5, FC3, FC1, FCz, FC2, FC4, FC6, C5, C3, C1, Cz, C2, C4, C6, within a 300-500 ms time window.

The analysis revealed a significant interaction effect of stimulus memory status and experiment in a left cluster comprising electrodes F5, FC5 and C5, between 301 ms and 480 ms (*p* < .001, spatial peak at electrode C5, temporal peak at 402 ms, **Figure S1A**). The average amplitudes and topographies extracted from this cluster showed a difference in the old/new ERP modulations between the two experiments (**Figure S1B, C**). Specifically, in the font-association group new stimuli exhibited enhanced amplitudes compared to old ones across the entire ROI time window. In contrast, in the character-association group, amplitudes were comparable between memory status conditions.


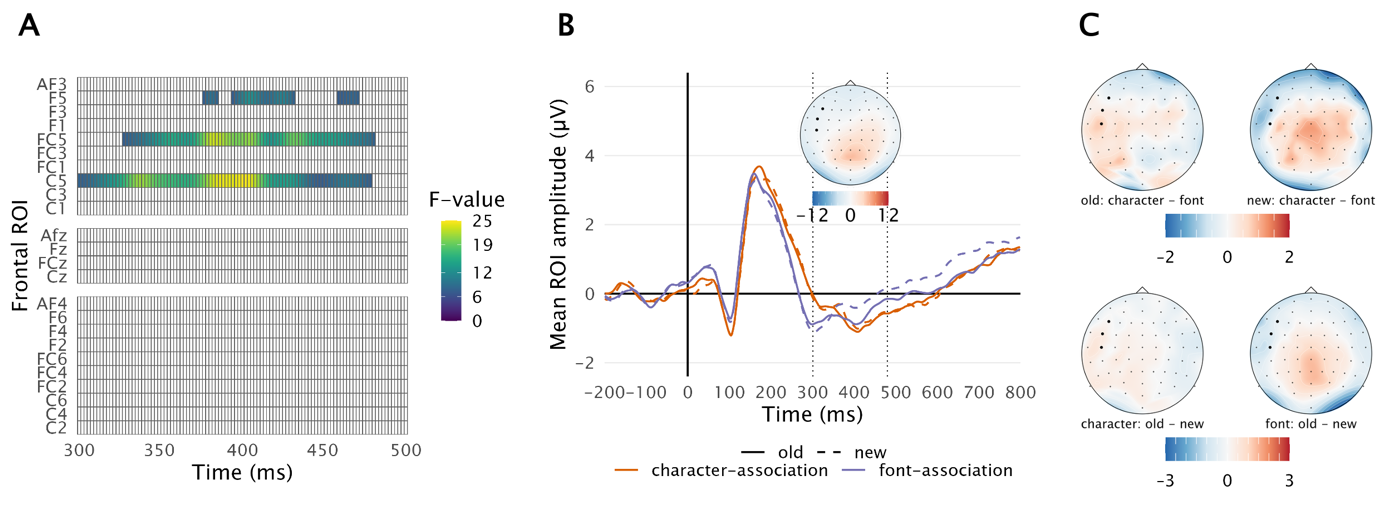


**Figure S1**. ERP modulations by stimulus memory status and experiment at frontal electrode sites. **(A)** Significant cluster of interaction effects of stimulus memory status and experiment in the predefined spatiotemporal ROI. **(B)** Grand-averaged ERPs at the significant cluster’s electrodes, with corresponding scalp topography of grand-averaged ERP across all conditions. Dotted lines represent the temporal extent of the significant cluster. **(C)** Scalp topographies averaged across the time window of the significant cluster, contrasted for the two experimental groups, for old and new stimuli separately (top) and for old and new stimuli, for the two experiments separately (bottom).
